# The role of oxidative stress in spinal cord ischemia reperfusion injury: mechanisms and therapeutic implications

**DOI:** 10.3389/fncel.2025.1590493

**Published:** 2025-06-24

**Authors:** Yu Xing, Yuan-zhang Xiao, Min Zhao, Jiang-jun Zhou, Kai Zhao, Chun-lin Xiao

**Affiliations:** 1 Department of Orthopaedics, Frist Affiliated Hospital of Gannan Medical University, Ganzhou, Jiangxi, China; 2 Gannan Medical University, 1 Harmony Avenue, Rongjiang New District, Ganzhou, Jiangxi Province, China; 3 Department of Spine Surgery, Yingtan People’s Hospital, Yingtan, Jiangxi, China; 4 Hospital 908, Joint Logistics Support Force, Nanchang, Jiangxi, China

**Keywords:** spinal cord ischemia/reperfusion injury (SCIRI), oxidative stress, inflammation, cell death, mechanism

## Abstract

Spinal cord ischemia/reperfusion injury (SCIRI) is a serious disease that leads to the loss of sensory and motor functions and is a common complication after spinal cord injury, spinal cord degeneration or thoracic and abdominal aortic surgery. At present, the spinal cord is mainly protected from ischemic injury through treatment strategies such as hypothermia, surgery and drug assistance, but these intervention measures cannot effectively improve these conditions. SCIRI is a complex process that leads to cell damage and death. Among them, oxidative stress is an important pathological event of ischemia/reperfusion injury. Oxidative stress can initiate multiple inflammatory and apoptotic pathways, triggering a series of destructive events such as inflammatory responses and cell death, further deteriorating the microenvironment at the injured site, and leading to neurological dysfunction. Based on the important role of oxidative stress in SCIRI, we believe that targeted inhibition of oxidative stress responses can effectively reduce secondary injuries caused by trauma, which has a certain positive effect on the rehabilitation and prognosis of patients with SCIRI. This review systematically expounds the spatiotemporal dynamic characteristics of oxidative stress during the SCIRI process and its molecular regulatory network, with a focus on analyzing the multivariate generation mechanism of ROS. To deeply explore the regulatory effects of ROS on pathological processes such as neuronal death, inflammatory response and blood-spinal barrier disruption under SCIRI conditions, as well as its interaction patterns with signaling pathways. In order to form a systematic treatment for SCIRI caused by oxidative stress and promote the recovery of neurological function after injury. This review is helpful for us to understand the effect of oxidative stress on SCIRI and provides a theoretical basis for the treatment of SCIRI based on oxidative stress.

## 1 Introduction

Spinal cord ischemia/reperfusion injury (SCIRI) is characterized by the interruption of spinal cord blood flow, tissue hypoxia, and a severe impact on the normal metabolism and function of cells. However, after restoring blood perfusion, instead of restoring the spinal cord tissue and function, it triggers a series of complex and harmful pathophysiological reactions, leading to a highly destructive clinical condition that further aggravates spinal cord injury. It often occurs after acute spinal vascular events such as aortic dissection, thoracic and abdominal aortic aneurysm surgery, spinal surgery, and spinal vascular obstruction (Bell et al., 2013; Rong et al., 2022). After SCIRI, it can lead to severe sensory and motor dysfunction, including paralysis and paraplegia, causing patients to have limited movement, loss of autonomous activity ability, and even death (Ling et al., 2021; Smith et al., 2012). This not only causes great difficulties for patients to take care of themselves in daily life but also brings a heavy burden to families and society (O'shea et al., 2017). Spinal cord injury caused by ischemia–reperfusion injury can be divided into ischemic injury and reperfusion injury. Ischemic injury leads to hypoxia and nutrient deprivation, causing insufficient cellular energy and accumulation of metabolic by-products. During reperfusion, oxidative stress, inflammatory responses, and increased production of reactive oxygen species (ROS) may occur, thereby exacerbating secondary injury (Duan et al., 2024). At present, the treatment methods for SCIRI mainly focus on prevention, early identification, and intervention, including intraoperative neurological function monitoring, reducing spinal cord ischemia time, increasing spinal cord perfusion pressure, and enhancing the tolerance of the spinal cord to ischemia through hypothermia, etc., attempting to protect the spinal cord from ischemic injury, reduce the degree of injury, and promote neurological function recovery (Wynn and Acher, 2014; Hnath et al., 2008; Khachatryan et al., 2022). However, the effects of these intervention measures are often unsatisfactory, and it is difficult to fundamentally solve the problem of nerve injury (Lemaire et al., 2012).Therefore, in-depth exploration of the pathogenesis of SCIRI and the search for new treatment strategies have tremendous clinical significance. The pathological cascade of SCIRI involves the synergistic effect of multi-dimensional pathological mechanisms. Its development process covers key pathological links such as inflammatory cascade activation, programmed cell death (including apoptosis and autophagy), disruption of the integrity of the blood-spinal barrier (BSCB), imbalance of redox homeostasis, intracellular calcium ion homeostasis disorder, and excitatory amino acid neurotoxicity (Liu et al., 2018; Zhao et al., 2018).Particularly worthy of attention is that oxidative stress, as the core regulatory hub of this pathological network, plays a key driving role in SCIRI by mediating the imbalance of free radical metabolism (Xu et al., 2023). Specifically, the outbreak of ROS triggered by ischemia–reperfusion can induce a lipid peroxidation chain reaction. Through multiple mechanisms such as activating calcium-dependent proteases, promoting the release of pro-inflammatory factors, and enhancing the sensitivity of the mitochondrial apoptotic pathway, it eventually leads to the disintegration of neuronal structure and decompensation of spinal cord conduction function, resulting in irreversible neurological deficits (Yu et al., 2016; Al Mamun et al., 2021; He et al., 2021). Based on this, the microenvironment regulation of key nodes of oxidative stress (such as eliminating excessive ROS and repairing the antioxidant defense system) has been established as an important neuroprotective strategy for improving the prognosis of SCIRI, and its intervention effect directly affects the repair process and neurological functional outcome of the injured tissue (Sun et al., 2021).

Oxidative stress refers to the imbalance between the oxidation and antioxidant systems in the body when the body is exposed to various harmful stimuli, resulting in the excessive generation of ROS, such as superoxide anions, hydroxyl radicals, and hydrogen peroxide, which exceeds the body’s own antioxidant defense capacity (Pizzino et al., 2017; Boyle et al., 1999). Under physiological conditions, ROS as an inevitable by-product of cellular aerobic metabolism, maintains a precise dynamic balance with the endogenous antioxidant system (Farías et al., 2017). However, during ischemia–reperfusion, bleeding and fluid resuscitation can promote excessive production of ROS, leading to the decompensation of the antioxidant defense system and ultimately inducing a cascade of oxidative stress responses (Yu et al., 2019). The pathological process of SCIRI shows significant biphasic characteristics: During the acute ischemic period, neuronal overexcitation and glutaminotropic excitotoxicity activate the inflammatory cascade reaction, trigger the initial oxidative stress event, and promote the excessive generation of ROS. In the subsequent reperfusion stage, with the sudden increase in oxygen partial pressure, the dysfunction of the mitochondrial electron transport chain will directly induce the explosive generation of oxygen free radicals (Goldhaber and Weiss, 1992). This persistent oxidative damage can trigger various cellular structural damages, including but not limited to lipid peroxidation chain reactions, DNA oxidative damage, and pro-inflammatory factor storms, ultimately leading to cell death and irreversible neuronal damage (Li et al., 2019; Yu et al., 2023; Zhang et al., 2024). Recent studies have confirmed that oxygen free radicals produced during the acute phase of SCIRI are a key driver of secondary neuronal necrosis. They can aggravate the injury of spinal cord tissue through pathways such as activating inflammatory responses and inducing apoptosis, forming a vicious cycle that continuously amplifies the injury effect (Hall, 2011). Preclinical studies have shown that antioxidant therapy has demonstrated in animal models the preliminary evidence of effectively improving spinal cord nerve function by neutralizing excessive ROS, inhibiting neuronal apoptosis, regulating calcium homeostasis imbalance and alleviating inflammatory cascade reactions (Hall, 2011; Zhou et al., 2018; Han et al., 2020; Feng et al., 2021). However, it should be emphasized that these findings are limited to laboratory research. Current clinical data show that antioxidant therapy has not yet confirmed a clear neurorepair effect in human trials. Furthermore, the formation of oxidative stress after ischemia–reperfusion and its impact on spinal cord tissue injury involve multiple complex mechanisms. In view of the complexity of this mechanism, targeted regulation of oxidative stress after SCIRI has potential research value.

This review systematically expounds the spatiotemporal dynamic characteristics of oxidative stress during the SCIRI process and its molecular regulatory network, and analyzes the multiple generation mechanisms of ROS mediated by SCIRI (including mitochondrial electron transport chain leakage, Phosphoramide adenine dinucleotide (NADPH) oxidase activation, xanthine oxidase system imbalance, etc.), and explores in depth the interaction mode between ROS and Nrf2/ARE, NF-κB, MAPK and other signaling pathways under SCIRI pathological conditions, as well as their regulatory effects on pathological processes such as neuronal death, inflammatory response, and blood–brain barrier disruption. By integrating various intervention measures targeting the regulation of redox balance in recent years, it aims to form a systematic treatment for SCIRI caused by oxidative stress and promote neurological function recovery after injury. This review is helpful for us to understand the effect of oxidative stress on SCIRI and provides a theoretical basis for the treatment of SCIRI based on oxidative stress.

## 2 Literature retrieval strategy

On January 20, 2025, the first author initiated a search of multiple databases, covering literature published from March 1998 to January 2025. The accessed databases include China National Knowledge Infrastructure, Wanfang Database and PubMed. The search strategy uses keywords such as “spinal cord ischemia–reperfusion injury,” “mitochondria,” “activated oxygen,” “oxidative stress” and “inflammation” to establish selection criteria. Various combinations of these keywords were used during the retrieval process. The retrieved literature mainly includes original research papers, review papers, comments, case reports and meta-analyses. The inclusion criteria include studies related to oxidative stress and SCIRI, which are highly original, have clear conclusions, and have been published in well-known journals within the past 20 years. Exclusion criteria include studies that are irrelevant to the topic of this review or studies with repetitive content. After the initial search, the relevance to this study was determined by reviewing the title and abstract, and then the full text was read comprehensively to evaluate the central topic of the selected study.

## 3 Sources of ROS in SCIRI

Under normal physiological conditions, ROS is constantly produced in the body, and there is also a complete antioxidant defense system to maintain the dynamic balance of ROS. ROS is a type of oxygen-containing molecules with high chemical reactivity, mainly including singlet oxygen, superoxide anions (O_2_^-^), hydrogen peroxide (H_2_O_2_), and hydroxyl radicals (OH^−^) (Rahal et al., 2014; Giorgio et al., 2007). Oxidative stress is a series of adaptive responses caused by the imbalance between ROS levels and the antioxidant system. These responses lead to the accumulation of excessive free radicals and subsequent cytotoxic effects (Sies and Jones, 2020). ROS plays a crucial role in the occurrence and development of ischemia–reperfusion (I/R) injury (Halladin, 2015). After ischemia–reperfusion, ROS in the spinal cord is mainly produced by enzymatic reactions such as mitochondrial respiratory chain, xanthine oxidase, NADPH oxidase.

Mitochondria are the centers of intracellular energy metabolism and are also regarded as the main sites where ROS is produced (Quinlan et al., 2013). Under physiological conditions, the mitochondrial respiratory chain achieves energy conversion through a precisely coupled oxidative phosphorylation process. Specifically, the reduction equivalent released by the substrate dehydrogenation reaction is gradually transferred through the electron transport system composed of complex I, complex III and complex IV. Eventually, under the catalysis of complex IV, molecular oxygen undergoes electron reduction to form water molecules. In this cascade reaction process, the proton gradient across the inner mitochondrial membrane is gradually formed through the proton pump function established by complexes I, III, and IV, and drives the ATP synthase (complex V) to catalyze the phosphorylation of ADP to generate ATP, achieving an accurate coupling of redox reactions and energy storage (Zotta et al., 2024). However, during this process, some electrons escape from the respiratory chain, resulting in incomplete reduction of molecular oxygen, namely the generation of ROS (Li et al., 2022). The creation of ROS happens through oxidative phosphorylation, which is part of aerobic respiration in the mitochondrial electron transport chain (ETC). Mitochondrial ETC generates superoxides and H₂O₂ by regulating the proton gradient within the mitochondria (Cadenas, 2018). Electrons from complex 1 can directly react with oxygen to generate O₂^−^, rather than being reduced to H₂O (Brand, 2010). O_2_^−^ can be spontaneously or catalyzed by superoxide dismutase (SOD) to be converted into H₂O₂ and O₂ (Breton-Romero and Lamas, 2014). And H₂O₂ oxidizes Fe^2+^ to Fe^3+^ through the Fenton reaction to generate hydroxyl radicals (Kevin et al., 2005). However, under ischemic and hypoxic conditions, the electron transport in the mitochondrial respiratory chain is blocked. Some electron carriers in the electron transport chain, such as coenzyme Q, will directly transfer electrons to oxygen, causing oxygen molecules to undergo single-electron reduction and thereby generating a large amount of O_2_^−^. In addition, during the ischemic period, intracellular ATP production is significantly reduced, and the functions of ATP-dependent ion pumps are impaired, such as sodium-potassium pumps and calcium pumps, which cannot work normally. This leads to a large accumulation of Na- and Ca^2+^ within cells and an imbalance in the intracellular environment. The increase of intracellular calcium ion concentration will activate a series of enzymes, such as phospholipase A2, protease, etc. The activation of phospholipase A2 can decompose phospholipids on the cell membrane, generating metabolic products such as arachidonic acid, which further participate in inflammatory responses and the generation of ROS. The activation of proteases can degrade intracellular proteins, disrupt the normal structure and function of cells, and may also indirectly promote the production of ROS (Alizadeh et al., 2019).

During the reperfusion period, with the re-supply of oxygen, the xanthine oxidase system is activated, which is an important reason for the further intensification of oxidative stress (Neri et al., 2015). The xanthine oxidase system consists of two interconvertible forms: xanthine dehydrogenase (XDH) preferentially uses NAD^+^ as the electron acceptor, while xanthine oxidase (XO) uses O_2_ as the terminal electron acceptor (Granger and Kvietys, 2015). During the ischemic period, due to the reduced production of ATP, a large amount of hypoxanthine, an energy metabolite within cells, accumulates. During reperfusion, the supply of oxygen enables xanthine dehydrogenase to be rapidly converted into xanthine oxidase. Xanthine oxidase uses hypoxanthine and xanthine as substrates. With the participation of oxygen, it oxidizes them to uric acid and simultaneously generates a large amount of superoxide anions (Mccord, 1985; Reimer and Jennings, 1985). This process leads to a sharp increase in the generation of ROS, exceeding the antioxidant defense capacity of the organism itself.

**Figure 1:**
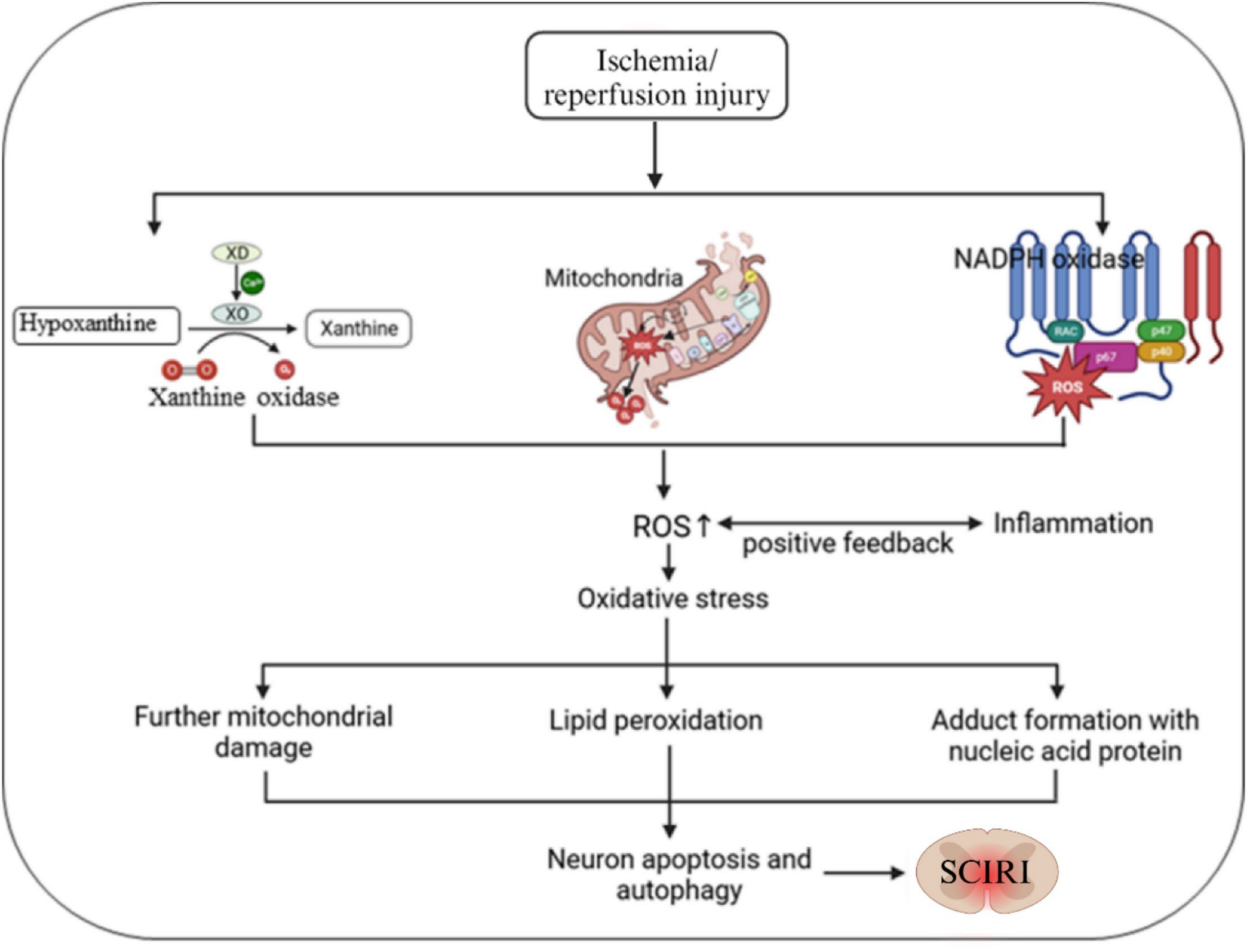
Schematic diagram of the generation mechanism of ROS during the SCIRI process. ROS is mainly produced through three pathways: mitochondria, xanthine oxidase, and NADPH oxidase. Oxidative stress injury occurs when the release of ROS exceeds the clearance capacity. Elevated ROS levels can induce lipid peroxidation, protein and DNA damage, and cross-interference in inflammatory responses, leading to further deterioration of SCIRI.

Phosphoramide adenine dinucleotide oxidase is a transmembrane protein complex and one of the important ROS-producing enzymes in the body. During ischemia–reperfusion, inflammatory cells such as neutrophils and macrophages are activated and, attracted by chemokines, gather at the site of ischemia–reperfusion injury (Taoka et al., 1997; Trivedi et al., 2006). Meanwhile, after being activated, these cells can activate NADPH oxidase. In the activated state, it uses NADPH as an electron donor to reduce oxygen to ROS, such as O₂^−^. These ROS can attack biological macromolecules such as cell membranes, proteins, and nucleic acids, triggering lipid peroxidation, protein oxidative modification, and DNA damage. This further leads to the destruction of cell structure and function (Khayrullina et al., 2015; Busquets-Cortés et al., 2018; Figure 1).

## 4 The impact of oxidative stress on SCIRI

**Figure 2:**
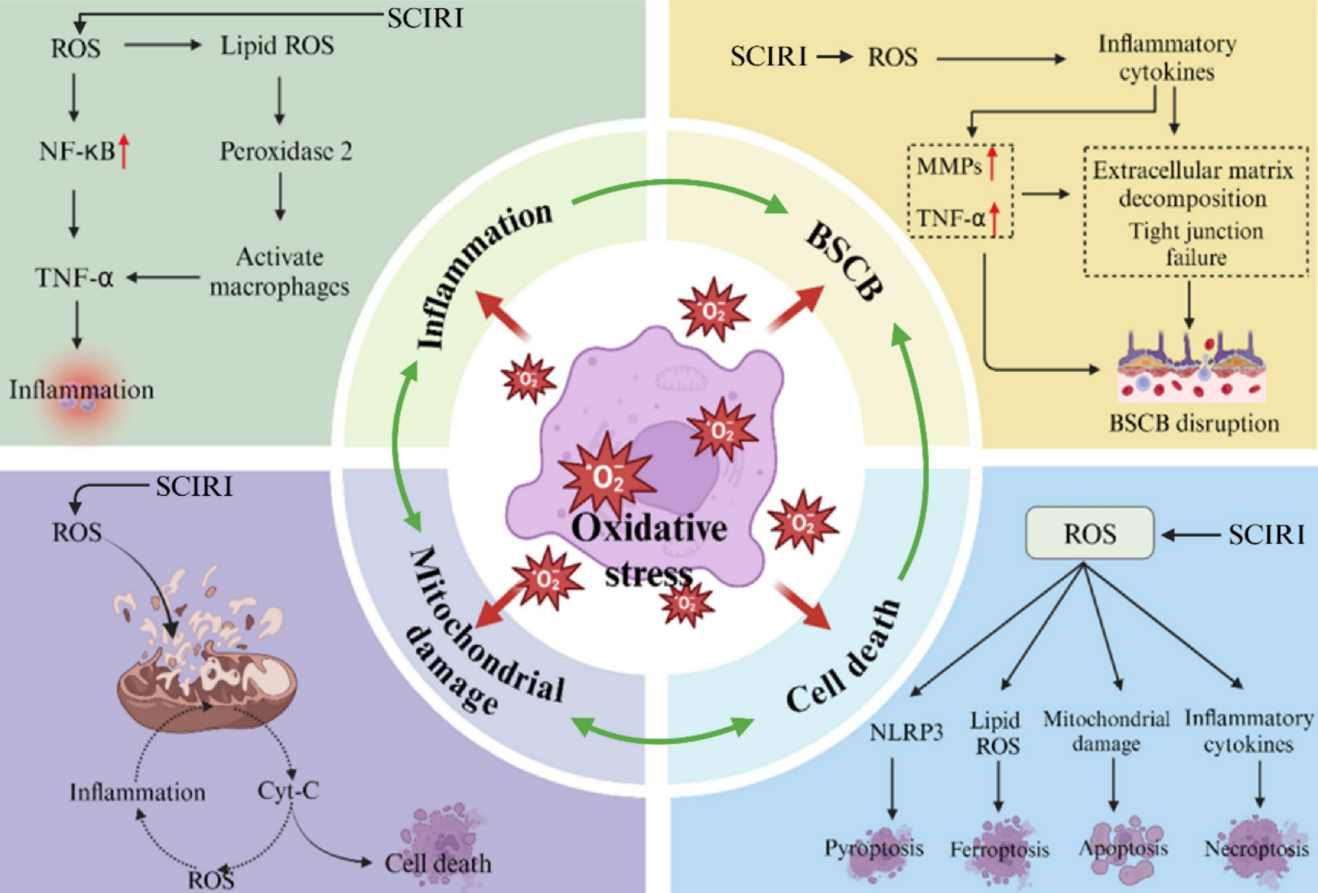
The effects of oxidative stress on inflammatory response, blood-spinal barrier, mitochondrial damage and cell death during SCIRI.

After SCIRI, a large amount of ROS can be produced, causing disorders in the body’s oxidation/antioxidant system, thereby leading to oxidative stress and oxidative damage (Gu et al., 2020; Khattab et al., 2022). During the spinal cord ischemia stage, a large amount of ROS is induced to be produced, activating macrophages to release pro-inflammatory mediators such as TNF-*α* and IL-1β, triggering neutrophil infiltration and inflammasome activation. Inflammatory factors induce the dissociation of ZO-1 protein from the tight junction complex, simultaneously up-regulate the expression of MMPs, directly degrade the collagen components of the basement membrane, and disrupt the integrity of BSCB. The disruption of BSCB triggers tissue edema caused by plasma extravasation, compressing microcirculation, and secondary infiltration of peripheral immune cells. This forms a cascade amplification effect of “inflammation-BSCB leakage-reinflammation” (Qu et al., 2009). Furthermore, ROS also activates various enzymes, including phospholipase A, thereby destroying the integrity and permeability of the cell membrane of the nervous system, leading to cell death and reperfusion injury (Chen et al., 2011; Hall et al., 2016). This three-dimensional pathological network spanning oxidative stress, inflammatory response, and barrier disruption provides a molecular basis for the irreversible nerve injury of SCIRI. In this section, we mainly discussed and described the negative effects of ROS during ischemia–reperfusion by regulating inflammatory responses, the blood-ridge barrier, mitochondrial damage, and programmed cell death (Figure 2).

### 4.1 Promote inflammatory responses

The inflammatory response is a defense response against exogenous and/or endogenous signals, but persistent inflammation has adverse effects on the body (Iantomasi et al., 2023). It is known that the inflammatory response plays an important role after ischemia and leads to spinal cord injury (Matsumoto et al., 2003). It was found through single-cell sequencing technology that inflammation-related immune cells infiltrated in SCIRI, including macrophages and neutrophils (Kim et al., 2019). These inflammatory cells produce many pro-inflammatory mediators, such as IL-1β, TNF-α, and IL-6, etc. These cytokines are important mediators of ischemic inflammatory responses in the spinal cord. They recruit peripheral inflammatory cells into the spinal cord and cause delayed neuronal death (Altinors, 2009; Ilhan et al., 2004). Oxidative stress is involved in the occurrence and development process of SCIRI and is also one of the important factors inducing inflammation (Fakhri et al., 2022). Inflammation in SCIRI can lead to apoptosis of neuronal cells and dysfunction of the blood–brain barrier, thereby accelerating the pathological process of SCIRI (Fu et al., 2018). The increase in ROS production can lead to protein oxidation, and protein oxidation is converted into the release of inflammatory signaling molecules, such as peroxidase 2 (PRDX2) (Berlett and Stadtman, 1997; Salzano et al., 2014). PRDX2 can also act as a redox dependent inflammatory mediator to activate macrophages to produce and release TNF- *α*, thereby altering the REDOX state of cell surface receptors and inducing inflammatory responses (Salzano et al., 2014). It is notable that the inflammatory response caused by oxidative stress can, in turn, enhance oxidative stress (Li et al., 2019). Mitochondria stimulated by TNF can increase ROS, leading to apoptosis and necrotic apoptosis, while ROS can promote the generation of TNF. A positive feedback loop is formed between the two (Blaser et al., 2016). The increase in ROS production at the injury site leads to the release of inflammatory factors and adhesion molecules, and mediates the inflammatory response by activating the NF-κB signaling pathway (Morgan and Liu, 2011). This circuit was also found during the process of spinal cord ischemia–reperfusion (Gökce et al., 2016). This indicates that during the process of spinal cord injury, inflammation and oxidative stress promote each other, eventually leading to a vicious cycle.

### 4.2 Damage the blood-spinal barrier

The blood-spinal cord barrier (BSCB) is usually regarded as an extension of the blood–brain barrier. The BSCB is composed of capillary endothelial cells and their accompanying basement membrane, pericytes, and astrocyte endopods. Its function is to regulate blood flow in cerebrospinal fluid and control the inflow and outflow of nutrients, energy storage, metabolites, and toxins within the neurovascular unit (Mcconnell et al., 2017; Li et al., 2017). Some studies reported that increased levels of superoxide contribute to BSCB endothelial dysfunction (Lochhead et al., 2010). Oxidative stress causes the release of several pro-inflammatory cytokines, which break down the extracellular matrix and damage proteins that help keep the blood–brain barrier intact, leading to its failure and changes in blood flow (Allen and Bayraktutan, 2009; Janyou et al., 2017). Furthermore, oxidative stress can also activate various redox sensitive transcription factors and participate in and accelerate the inflammatory response, leading to BSCB damage (Kim et al., 2022). Inflammatory cytokines are one of the most pivotal factors for BSCB disruption in SCIRI. They cause ZO-1 to separate from the cytoskeleton and increase the levels of MMPs and TNFα, which are often linked to higher BSCB permeability. Nevertheless, BSCB disruption in turn exacerbates inflammatory reactions, resulting in irreversible spinal cord damage (Ding et al., 2010).

### 4.3 Mitochondrial damage

Mitochondria, as the main target of intracellular oxidative stress, are highly susceptible to it in terms of their structure and function. First of all, ROS can directly attack the mitochondrial inner membrane system. Excessive accumulation of ROS can induce structural disorders (swelling or rupture) of the mitochondrial cristae and promote abnormal opening of the mitochondrial permeability transition pore (mPTP). The continuous opening of mPTP can cause swelling of the mitochondrial matrix, which in turn leads to mechanical rupture of the outer membrane and causes membrane potential (ΔΨm) collapse. Meanwhile, pro-apoptotic factors such as cytochrome C (Cyt C) are released into the cytoplasm, activating the Caspase-9/3 cascade reaction and ultimately initiating programmed cell death (Holzerová and Prokisch, 2015; Yang et al., 2015; Skulachev, 1998). Secondly, ROS interferes with the mitochondrial dynamic balance. ROS disrupts the homeostasis regulation of mitochondrial division and fusion by oxidizing and modifying fissile proteins or fusion proteins, resulting in excessive fragmentation of the mitochondrial network. Fragmented mitochondria, due to the increase in surface area and the disorder of electron transport chains, further intensify the abnormal generation of ROS, thereby forming a vicious cycle and amplifying oxidative stress (Bernardi et al., 2015). Furthermore, damaged mitochondria amplify apoptotic signals through multiple pathways. In addition to releasing Cyt C, the rupture of its outer membrane can also lead to the outflow of pro-apoptotic proteins. By inhibiting apoptotic inhibitory proteins or directly cutting DNA, it synergistically enhances the caspase-dependent and non-dependent cell death pathways (Anne Stetler et al., 2013).

### 4.4 Cell death

Cell death is a highly conserved biological process, which not only participates in the morphogenesis and developmental regulation of organisms, but also plays an important role in various pathophysiological processes. Different cell death patterns exhibit unique morphological characteristics and molecular mechanisms, and mediate the pathogenesis of diseases through different pathways (Galluzzi et al., 2018). Apoptosis is a process of cell death that occurs through the activation of caspase cascade apoptosis under the strict regulation of apoptosis genes. It is characterized by cell contraction, nuclear condensation and chromatin condensation, and eventually forms apoptotic bodies (Tang et al., 2019; Maiuri et al., 2007). Pyroptosis is a type of pro-inflammatory cell death that depends on the activation of caspase-1 and the formation of plasma membrane pores by gasdermin-D (GSDMD) (Bergsbaken et al., 2009). Ferroptosis is a unique non-apoptotic regulated cell death, whose main characteristics are abnormal iron metabolism and lipid peroxidation within cells (Dixon et al., 2012). Necroptosis is a programmed form of cell death that does not rely on caspase (Zhu et al., 2022). In addition, other cell death patterns also exhibit unique mechanisms: Necrosis, as a non-programmed passive death, is often triggered by acute injury, accompanied by swelling of organelles and rupture of the plasma membrane (D'arcy, 2019). Parthanatos is a form of death caused by the excessive activation of PARP-1 activated by DNA damage, characterized by the release of mitochondrial apoptosis-inducing factors and chromatin dispersion (Huang et al., 2022). Autophagia mediates through excessive formation of autophagosomes and can induce cellular degradation under specific conditions (Sepehr et al., 2020). Entosis is a form of invasive cell death. Through cytoskeletal remodeling, target cells are phagocytosed by adjacent cells, and this process has special pathological significance in the tumor microenvironment (Mlynarczuk-Bialy et al., 2020). These diverse pathways of death constitute a complex regulatory network, which interact to jointly maintain tissue homeostasis and affect disease outcomes.

**Figure 3:**
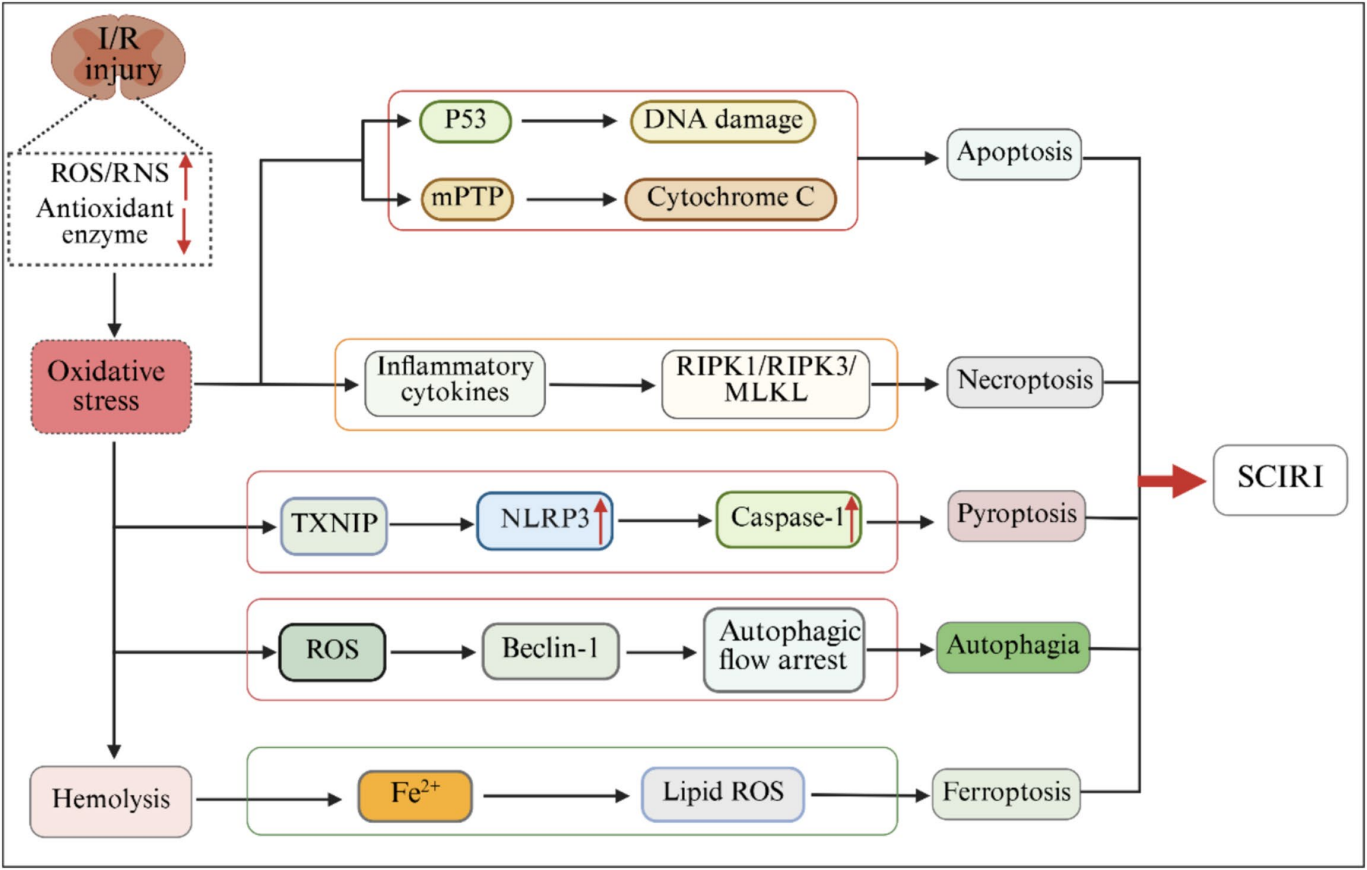
The regulatory mechanism of oxidative stress on cell death in SCIRI.

A large number of studies have confirmed that ROS plays an important role in the regulation of cell death, and it participates in the molecular cascade reactions of different death patterns (apoptosis, necrotizing apoptosis, pyroptosis, ferroptosis, and autophagy) through multiple pathways (Orrenius et al., 2007; Ryter et al., 2007; Figure 3). As a classic caspase-dependent programmed death, the initiation and execution of apoptosis are mainly regulated by caspase family proteases (Green, 1998). This process can be activated through two main pathways: exogenous and endogenous, and ROS, as a key regulatory hub, has an activation effect on both pathways (Mirzaei et al., 2021). In the endogenous apoptotic pathway, ROS induces the opening of mitochondrial mPTP by directly oxidizing the phospholipid components of the mitochondrial inner membrane (such as cardiolipin), promoting the release of Cyt C into the cytoplasm. Cyt C in the cytoplasm, together with apoptotic protease activator factor-1 (Apaf-1) and procaspase-9, jointly forms apoptotic body complexes. Through cascade activation of caspase-9 and its downstream effector proteins (such as caspase-3/7), it ultimately triggers apoptosis (Kalkavan and Green, 2018). In the exogenous apoptotic pathway, ROS promotes the formation of a death-inducing signal complex between adaptor protein FADD and procaspase-8/10 by activating death receptors such as TNF-R1, TRAIL-R1/2 and Fas. This complex activates caspase-8/10 and further activates downstream effector caspases (such as caspase-3/6/7), thereby initiating the apoptotic program and triggering apoptosis (Li et al., 1997). Necrotic apoptosis is a form of programmed necrosis regulated by genetic programming, and its core executive mechanism relies on the activation of the receptor-interacting protein kinase 1 (RIPK1) -RIPK3-MLKL signaling axis (Degterev et al., 2005; Zhou et al., 2022). This process can be initiated by various exogenous stimuli, including interferon, tumor necrosis factor and Toll-like ligands, etc. These stimuli eventually trigger the necrotic apoptosis cascade by activating the downstream death domain signal transduction network (Zhang et al., 2017). It is notable that ROS has been proven to mediate the execution of cell necrosis. Under oxidative stress conditions, the accumulated ROS in cells activates the RIPK1/RIPK3 complex, promotes the phosphorylation and oligomerization of mixed lineage kinase domain-like proteins, and enables their translocation to the cell membrane to form ion channels, thereby initiating the classic RIPK1-RIPK3-MLKL pathin-dependent necrotic apoptosis. Furthermore, under specific pathological conditions, ROS can also promote the release of apoptosis-inducing factors from mitochondria by activating polyadenosine diphosphate ribose polymerase, thereby triggering necrotizing apoptosis mediated by the non-classical RIPK1/RIPK3-PARP-AIF signaling axis (Mármol et al., 2017; Han et al., 2018). Pyroptosis is a caspase-1-dependent type of pro-inflammatory cell death, which differs from other forms of programmed cell death in terms of morphology and biochemical characteristics (Liu and Sun, 2019; Galluzzi et al., 2015; Fink and Cookson, 2006). Pyroptosis is divided into the classical inflammasome pathway mediated by caspase-1 and the non-classical pathways mediated by caspase 4, 5, and 11 (Lacey et al., 2018). Mitochondrial reactive oxygen species (mtROS) regulate pyroptosis by influencing lipid metabolism and inflammation. mtDNA oxidized by mtROS is released into the cytoplasm along with mitochondrial membrane damage, binding and triggering the activation of NLRP3 inflammatosomes, thereby inducing pyroptosis (Svensson et al., 2022; Zhong et al., 2018). It is reported that ROS is a common upstream mechanism in the activation process of NLRP3 inflammasome. ROS activates NLRP3 through the redox sensor thioredoxin interacting protein and triggers pyroptosis through the ROS/NLRP3-mediated caspase-1 pathway (Qiu et al., 2019; He et al., 2016). Ferroptosis is a form of cell death caused by the accumulation of iron-dependent lipid peroxides, and its main characteristics are abnormal iron metabolism and lipid peroxidation within cells (Dixon et al., 2012). During the SCIRI process, the red blood cells at the bleeding site rupture and release a large amount of free iron. Catalyzed by heme oxygenase-1 (HO-1), active iron ions are generated, resulting in local iron overload. Meanwhile, the activation of hypoxia-inducible factor (HIF-1α) promotes the outbreak of ROS and intensifies the excitotoxicity of glutamate by inhibiting the function of the glutamate-cystine reverse transporter. Multiple factors synergistically drive the occurrence of ferroptosis (Su et al., 2019). ROS weakens the cellular antioxidant defense system by consuming glutathione (GSH) and inhibiting the activity of glutathione peroxidase 4 (GPX4), leading to irreversible accumulation of lipid peroxides and ultimately causing ferroptosis (Yang et al., 2020). Autophagy is a highly conserved cellular self-degradation mechanism in eukaryotes. It degrades damaged or redundant intracellular components (such as abnormal proteins and senescent organelles) through lysosome-dependent pathways to maintain intracellular homeostasis and respond to stressful environments (Liu et al., 2023). In SCIRI, there exists a dynamic and bidirectional regulatory relationship between oxidative stress and autophagy, jointly influencing the fate choices of nerve cells (Yuan et al., 2023). Hypoxia during the ischemic period leads to dysfunction of the mitochondrial electron transport chain, triggering explosive accumulation of ROS. The recovery of oxygen supply during the reperfusion period further aggravates oxidative stress through pathways such as xanthine oxidase (Klionsky and Emr, 2000). Excessive ROS oxidizes and modifies key autophagy proteins (such as thiol oxidation of Beclin-1) and lysosomal membrane lipids, leading to autophagic flow arrest (such as autophagosome-lysosomal fusion obstruction), promoting abnormal accumulation of autophagic substrates and inducing autophagy-related death (Xie et al., 2017). The above studies indicate that ROS plays an important regulatory role in the cell death of SCIRI.

## 5 Signaling pathways involved in oxidative stress related SCIRI

**Figure 4:**
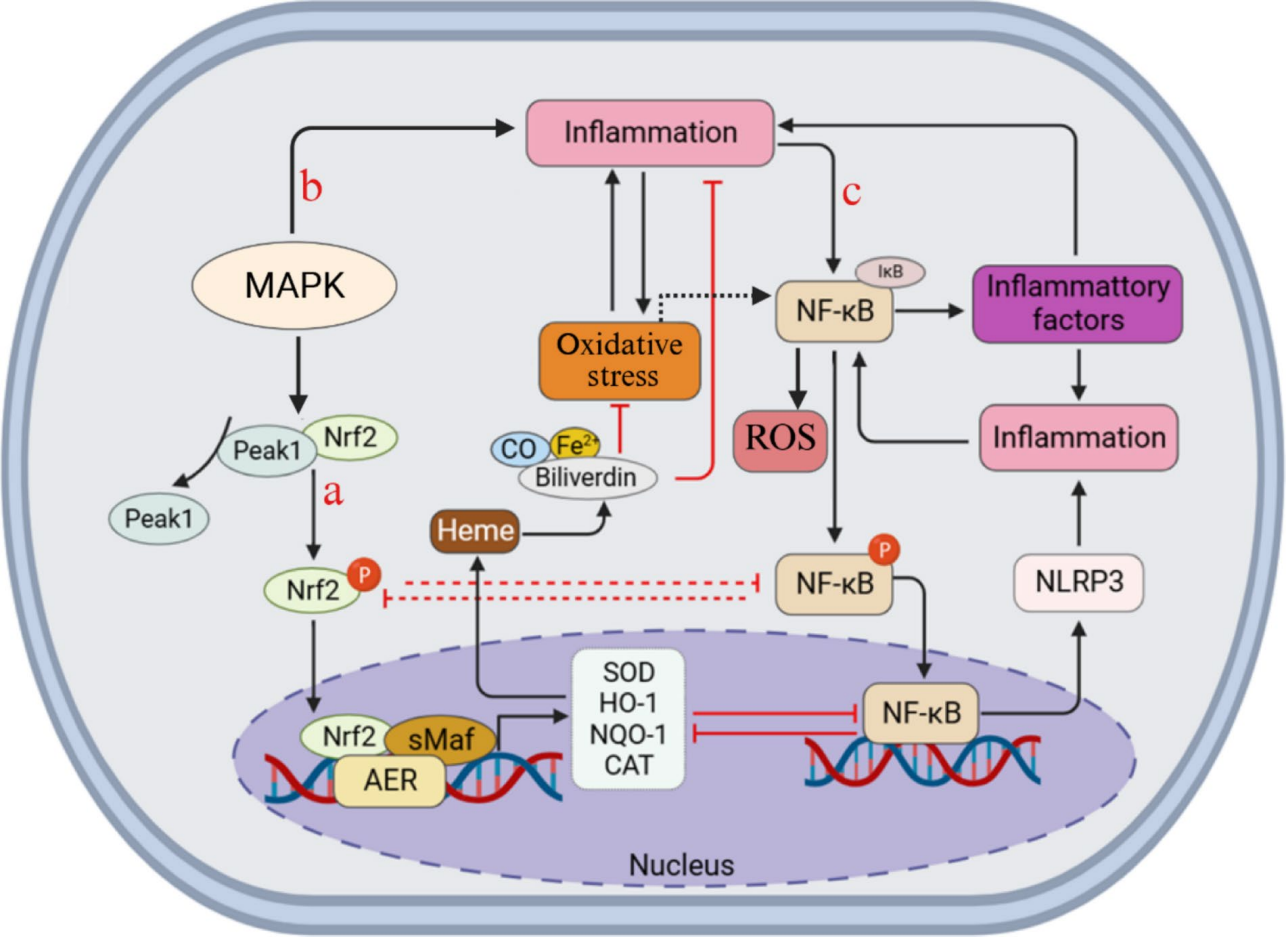
The oxidative stress process in SCIRI may involve signaling pathways and interaction mechanisms. After the MAPK pathway is activated, it promotes the dissociation of Nrf2 and Keap1. Nrf2 translocates to the nucleus, heterodimerizes with sMaf in the nucleus, and binds to an enhancer sequence called ARE. This, in turn, stimulates the expression of HO-1, NQO1, CAT, and SOD, thereby inhibiting oxidative stress through feedback. Nrf2 and NF-κB can influence each other and jointly regulate the pathological process.

Oxidative stress is an important pathological process in SCIRI and is regulated by multiple pathways, including signaling pathways such as Nrf2, NF-κB, and MAPKs. This chapter will introduce the role of these signaling pathways in oxidative stress in SCIRI (Figure 4).

### 5.1 Nrf2 pathway

Nuclear factor erythrocyte 2-related factor 2 (Nrf2) is a redox sensitive transcriptional regulatory factor, and its core function is to regulate the transcriptional activation of downstream genes by binding to antioxidant response elements (ARE) (Ma, 2013). Under normal physiological conditions, Kelch-like epichlorohydrin-1 (Keap1) continuously promotes the proteasome degradation of Nrf2 through the ubiquitination pathway and maintains its low expression level. When the imbalance of the body’s oxidation-antioxidant homeostasis leads to abnormal accumulation of ROS, the negative regulatory effect of Keap1 is released, thereby inhibiting the degradation process of Nrf2 (Lu et al., 2016). Under oxidative stress, Nrf2 dissociates from Keap1 and undergoes nuclear translocation. By forming heterodimer complexes with the small Maf protein in the nucleus, it specifically recognizes the enhancer region regulated by ARE. This further initiates the transcriptional activation of antioxidant/drug metabolism enzyme genes including HO-1, SOD, quinone oxidoreductase (NQO1), and glutathione peroxidase (GPX), etc. (Krajka-KuŹniak et al., 2017; Hu et al., 2016). Through the above cascade reaction, cells were able to enhance the activity of the antioxidant enzyme system, eliminate excessive ROS, and antagonize oxidative damage (Wang et al., 2012). Through the above cascade reaction, cells are able to enhance the activity of the antioxidant enzyme system, eliminate excessive ROS and antagonize oxidative damage. It is notable that HO-1, as an endogenous protective enzyme with consistently high expression in spinal cord injury, can persist in SCIRI and significantly alleviate oxidative stress injury by stabilizing mitochondrial function (Lin et al., 2016). Furthermore, after activation of Nrf2, it can also up-regulate the expression of the downstream target gene HO-1, and counteract oxidative stress by removing the strong oxidant heme and increasing the levels of endogenous antioxidants carbon monoxide and bilirubin (Prestera et al., 1995). Zhang et al. treated the rat SCIRI model with ginsenoside Rg1. They found that Nrf2 was activated. The activation of Nrf2 increased the enzymatic activities of SOD and GPx by inducing the expression of HO-1, and simultaneously inhibited the generation of lipid peroxidation marker MDA, thereby exerting an antioxidant effect (Zhang et al., 2022). It is particularly worth noting that NQO1 regulated by Nrf2, specifically catalyzes the conversion of quinone substances to hydroquinone through reduction reactions, effectively blocking the process of ROS generation by quinone compounds in the redox cycle. This mechanism plays a key role in alleviating secondary oxidative damage (Jung and Kwak, 2010). In addition, Nrf2 can also exert antioxidant effects by promoting the regeneration of oxidative cofactors and proteins. In the SCIRI rat model, melatonin promotes the reduction of glutathione disulfide by glutathione reductase by activating Nrf2 (Kryl'skii et al., 2019). Meanwhile, Nrf2 regulates the production of mitochondrial ROS by enhancing the biosynthesis of glutathione and the activity of glutathione reductase, up-regulating the enzymes in the pentose phosphate pathway, and regulating the expression level of mitochondrial antioxidant enzymes, thereby protecting mitochondria from oxidative damage (Ebrahimy et al., 2022; Chen et al., 2022). Furthermore, Nrf2 can also regulate the expression of HO-1 and NADPH quinone oxidoreductase-1 genes by directly acting on the ARE element, thereby inducing the expression of stress response proteins (Li et al., 2019; Figure 4a).

### 5.2 NF-kB pathway

Nuclear factor-κB (NF-κB) is a complex protein system composed of transcription factors and is an important nuclear transcription factor within cells. The NF- κB family consists of five members: RelA (p65), RelB, c-Rel, precursor proteins NF- κB1 (p105) and NF- κB2 (p100) (Oeckinghaus et al., 2011). Studies have confirmed that NF-κB signal transduction is activated after SCIRI (Jin et al., 2021). Oxidative stress is a powerful stimulating factor for the activation of NF-κB. The activated NF-κB is involved in the regulation of various pathological processes, including inflammatory responses, oxidative stress and apoptosis, etc. The research found that after SCIRI, increased levels of ROS and malondialdehyde (MDA), decreased activity of SOD, and accumulation of inflammatory factors (IL-1β and IL-6) and TNF-*α* were observed (Bao et al., 2016). The excessive ROS generated can directly activate NF-κB or indirectly activate NF-κB through redox factor-1 or other pathways, such as regulating the activity of IκB kinase or affecting the affinity between NF-κB and IκB, thereby further promoting the release of inflammatory factors and ROS (Nguyen et al., 2019; Angkeow et al., 2002; El Assar et al., 2013; Figure 4b). Furthermore, NF-κB is also a redox regulated transcription factor (Karin et al., 2004). Wang et al. used stearic acid chitosan nanoparticles loaded with sesamol to intervene in LPS-induced spinal motor neuron cells and found that NF-κB was significantly inhibited, leading to increased SOD and GPx activity and reduced release of ROS and inflammatory factors, thereby weakening oxidative stress and inflammatory damage (Wang et al., 2021).

### 5.3 MAPK pathway

Mitogen-activated protein kinase (MAPK) family is a conserved serine/threonine protein kinase family, consisting of extracellular signal-regulated kinase (ERK), p38 and c-Jun NH 2-terminal kinase (JNK) (Liu et al., 2020; Kanno et al., 2009). Each MAPK signal contains at least three parts: MAPK kinase kinase (MAP3K), MAPK kinase (MAP2K), and MAPK (Kim and Choi, 2010). The MAPK pathway is an important transmembrane signal transduction pathway, including MAPKs, ERK1/2, JNK, P38, etc. These pathways are relatively sensitive to ROS stimulation (Son et al., 2011). It is reported that the activation of MAPK is crucial for the production of various inflammatory cytokines and the regulation of oxidative stress (Bachstetter et al., 2011). During the pathological process of SCIRI, the level of ROS significantly increased (Ahuja et al., 2017). Excessive production of ROS can trigger oxidative stress, which then activates the p38 MAPK signaling pathway, thereby inducing oxidative stress and inflammatory responses, and leading to cell death (Bode et al., 2012; Huang et al., 2018). Under the stimulation of oxidative stress or other damage effects, the body produces cytokines such as interleukin and TNF-*α*. These cytokines can activate the MAPK signaling pathway and promote the nuclear translocation of Nrf2, thereby initiating antioxidant effects (Han et al., 2016; Zhao et al., 2016; Chi et al., 2015; Figure 4c).

## 6 Remote ischemic preconditioning after SCIRI

It is reported that remote ischemic preconditioning (RIPC) is an effective method for alleviating ischemia–reperfusion injury (Liang et al., 2019). RIPC is a method to protect distant target tissues/organs from subsequent fatal ischemic injury through intermittent ischemia–reperfusion of tissues/organs (Hausenloy and Yellon, 2011; Yu et al., 2016). In clinical practice, RIPC is usually achieved by applying periodic inflation-deflation to the upper or lower limbs of patients through a standard blood pressure cuff (Lee et al., 2023). The specific operation is to wrap the blood pressure cuff around one or both limbs (such as the upper arm or thigh), inflate until the pressure is higher than the systolic pressure, continuously block the blood flow for 5 min, then release the air for 5 min to resume perfusion. This process is repeated for 3 to 4 cycles (Lee et al., 2023). Preclinical data and clinical trials have shown that RIPC therapy can alleviate myocardial injury and improve the short-term prognosis of patients undergoing coronary artery bypass grafting (Kleinbongard et al., 2018). Meanwhile, RIPC can promote the release of molecules with damage-related molecular patterns, thereby activating the natural defense system and protecting the kidneys from subsequent inflammation and chemical damage (Gassanov et al., 2014). Some studies have shown that RIPC can protect the spinal cord from I/R injury (Yunoki et al., 2017). In the rat brain I/R injury model, researchers found that distal ischemic postconditioning could reduce the permeability of the BSCB and brain edema after cerebral ischemia, thereby reducing the infarct area and improving neurological outcomes (Li et al., 2015; Ren et al., 2011). This method has the characteristics of non-invasiveness, simplicity, convenience, repeatability and cost-effectiveness. Animal experiments have shown that RIPC can protect the spinal cord from I/R injury. Dong et al. found in a rabbit experiment that achieving RIPC through bilateral femoral artery occlusion (10 min of ischemia/10 min of reperfusion, 2 cycles) before aortic occlusion of spinal cord ischemia in rabbits could significantly improve neurological prognosis and alleviate spinal cord tissue damage. They also found that in the rat model, limb RIPC-induced spinal cord ischemic tolerance was achieved through tertiary circulation of the right femoral artery and 3-min ischemia/3-min reperfusion (Dong et al., 2010; Su et al., 2009). Previous studies have shown that RIPC can reduce oxidative stress during cardiopulmonary bypass, thereby alleviating nerve injury (Arvola et al., 2016; Bystrom et al., 2017). In the oxygen–glucose deprivation experiment simulating cerebral ischemia, RIPC exerts anti-apoptotic and antioxidant effects in neural stem cells, thereby protecting human neural stem cells from cell death (Motomura et al., 2017). Meanwhile, Gu et al. also found that RIPC could promote the recovery of neurological function in SCIRI mice, inhibit spinal cord oxidative stress, and reduce neuronal damage (Gu et al., 2023). These studies show that RIPC can protect the spinal cord from I/R injury.

## 7 Therapy target for oxidative stress related SCIRI

During I/R injury, a large amount of ROS is generated around the injury site, which can cause excessive oxidative stress, leading to neuronal death and accelerating the development of SCIRI. It is notable that reducing oxidative stress may be a promising approach for the treatment of SCIRI (Yu et al., 2019) (Table 1).

**Table 1:** Potential regulatory modes and regulators of oxidative stress in SCIRI treatment.

Regulation modes/regulator	Mechanisms	Models	Effect	References
Natural compound				
Curcumin	LDH↓, MDA↓, SOD↑, GSH-Px↑ → Inhibit oxidative stress	*In vivo/in vitro*	Anti-oxidant stress	Wu et al. (2024)
Salidroside	ROS ↓ → Inhibition of mitochondrial damage → Inhibit oxidative stress → Inhibit apoptosis	*In vivo*	Antioxidant stress and inhibit apoptosis	Gu et al. (2020)
Chickpea A	Nrf2/HO-1↑ → ROS ↓ → Inhibit oxidative stress	*In vivo*	Anti-oxidant stress	Li et al. (2024)
Astaxanthin	SOD↑, GSH-Px↑ → Inhibit oxidative stress → Promote the recovery of nerve function	*In vivo*	Antioxidant stress	Fu et al. (2020)
Ginsenoside Rb1	MDA↓, SOD↑ → Inhibit oxidative stress →Inhibit apoptosis	*In vivo*	Antioxidant stress and inhibit apoptosis	Ye et al. (2019)
Perillaldehyde	MDA↓, SOD↑, GSH-Px↑ → Inhibit oxidative stress	*In vivo/in vitro*	Anti-oxidant stress	Zheng et al. (2021)
Luteolin	MDA↓, SOD↑, GSH-Px↑ → Inhibit oxidative stress	*In vivo*	Anti-oxidant stress	Fu et al. (2018)
Syringaldehyde	MDA↓, SOD↑, GSH-Px↑ → Inhibit oxidative stress	*In vivo/in vitro*	Anti-oxidant stress	Malçok et al. (2020)
Pharm-aceuticals				
Dexmed-etomidine	MDA↓, SOD↑, GSH-Px↑ → Inhibit oxidative stress	*In vivo*	Anti-oxidant stress	Liu et al. (2023)
Lamotrigine	lipid peroxidation↓, SOD↑, GSH-Px↑ → Inhibit oxidative stress	*In vivo*	Anti-oxidant stress	Kahveci et al. (2021)
Atorvastatin	MDA↓, SOD↑, GSH-Px↑ → Inhibit oxidative stress	*In vivo*	Anti-oxidant stress	Kardes et al. (2017)
Rosuvastatin	MDA↓, SOD↑, GSH-Px↑ → Inhibit oxidative stress	*In vivo*	Anti-oxidant stress	Yavuz et al. (2013)
Simvastatin	MDA↓, SOD↑, GSH-Px↑ → Inhibit oxidative stress	*In vitro*	Anti-oxidant stress	Sohn et al. (2017)
Other				
Hyperbaric oxygen	SOD↑, GSH-Px↑ → ROS↓ → Inhibit oxidative stress	*In vivo*	Anti-oxidant stress	Nie et al. (2006)
Acupuncture	SOD↑, GSH-Px↑ → ROS↓ → Inhibit oxidative stress	*In vivo*	Anti-oxidant stress	Cai and Shen (2018)

### 7.1 Native compound

In recent years, the treatment of spinal cord injury using monomer compounds of traditional Chinese medicine has received extensive attention. Curcumin is a known active component in turmeric. Due to its role in scavenging free radicals, it has been widely studied in I/R traumatic diseases (Mokhtari-Zaer et al., 2019; Machado et al., 2022). For example, renal ischemia/reperfusion injury (Machado et al., 2022), cardiac I/R injury (Mokhtari-Zaer et al., 2019). In studies related to SCIRI, it was found that curcumin can alleviate neuronal apoptosis and oxidative stress induced by OGD/R by inhibiting oxidative markers LDH and MDA and increasing the levels of antioxidant stress SOD and GSH-Px, thereby slowing down the progression of SCIRI (Wu et al., 2024). Salidroside (Sal) is an effective component extracted from *Cistanche deserticola* and has multiple biological properties such as antioxidation, anti-apoptosis and neuroprotection (Cui et al., 2015). Gu research team confirmed in both *in vivo* and *in vitro* experiments of SCIRI that Sal can reduce the abnormal generation of ROS through antioxidant stress, lower the abnormal opening of mPTP, alleviate mitochondrial structural damage, and inhibit the release of Cyt C, blocking the activation of the mitochondrial-dependent apoptotic pathway (Gu et al., 2020). Nrf2 is a protective transcription factor that can regulate the oxidative stress response in cells, while HO-1 is an antioxidant protease that can enhance the resistance of neuronal cells to oxidative stress and protect neurological function (Abdel-Wahab et al., 2020). Hummus A (BA) is a methoxy isoflavone with antioxidant, anti-inflammatory and anti-apoptotic effects (Guo et al., 2019). A study has shown that BA alleviates cerebral ischemia/reperfusion injury by inhibiting apoptosis, endoplasmic reticulum stress and mitogen-activated protein kinase 14 pathway, thereby serving as an effective neuroprotective agent (Guo et al., 2021). In the SCIRI model, experimental studies have shown that BA intervention can specifically activate the Nrf2-mediated antioxidant defense system, significantly up-regulate the expression level of HO-1, and thereby effectively antagonize the oxidative stress cascade reaction induced by I/R, thereby significantly delaying I/R damage (Li et al., 2024). Astaxanthin (AST) is a powerful antioxidant and anti-inflammatory agent with multi-target neuroprotective effects (Fakhri et al., 2019). In the rat model of SCIRI, AST can alleviate pathological tissue damage and improve neurological function recovery after SCIRI by reducing oxidative stress (Fu et al., 2020). Ginsenoside Rb1 (GRb1) belongs to ginsenoside diol and has antioxidant and anti-apoptotic effects (Wynn et al., 2014). Previous studies have shown that GRb1 can significantly alleviate ischemia–reperfusion injury of the kidneys, brain, etc. (Schroeder et al., 2014). In the SCIRI study, GRb1 pretreatment of SCIRI rats can reduce neuronal apoptosis by increasing SOD activity in serum and spinal cord tissues and reducing the increase in MDA content. This indicates that GRb1 can alleviate SCIRI -induced apoptosis by inhibiting oxidative stress (Ye et al., 2019). In addition, monomer compounds such as Perilla aldehyde (Zheng et al., 2021), luteolin (Fu et al., 2018), and syringaldehyde (Malçok et al., 2020) can delay spinal cord I/R injury by reducing oxidative stress in ischemic spinal cord.

However, although these monomer compounds of traditional Chinese medicine have shown potential in basic research, their clinical transformation still faces multiple challenges. Firstly, the pharmacokinetic data of most compounds are limited, including that the absorption, distribution, metabolism and excretion characteristics have not been fully clarified, resulting in the lack of an optimization basis for the dosing regimen. Secondly, the high selectivity of the blood-spinal cord barrier severely limits the enrichment of drugs at the injury site. How to improve the targeted permeability remains a technical difficulty. Furthermore, most of the existing studies are based on animal models. The equivalence between the experimental dose and the human therapeutic dose is not yet clear, and the dose standards are inconsistent in different studies, which may affect the repeatability of therapeutic effects. More importantly, the vast majority of current achievements are still at the preclinical stage, lacking systematic clinical trials on human safety and efficacy, making it difficult to assess their actual medical value. In the future, strategies such as nano-delivery systems and structural modifications need to be adopted to improve bioavailability, and a standardized treatment system should be established in combination with multi-center clinical trials to promote the true clinical application of these natural active ingredients.

### 7.2 Pharmaceuticals

The recovery of neurological function after injury is a long process accompanying the entire pathological process. It has been found that many drugs can delay injury and promote the recovery of neurological function by inhibiting oxidative stress. For example, dexmedetomidine as a selective *α* 2-adrenergic receptor agonist, due to its extensive physiological functions and biological activities, multiple studies have shown that dexmedetomidine has a beneficial effect on I/R injury (Bunte et al., 2020). In the SCIRI rabbit model, dexmedetomidine can reduce the MDA content in spinal cord tissue, increase the SOD content, and improve the oxidative stress state, thereby alleviating SCIRI in rabbits (Liu et al., 2023). Lamotrigine is an anticonvulsant drug with multi-ion channel inhibitory properties, which has been proven to alleviate secondary neuronal injury by influencing different pathways. In studies related to SCIRI, Kahveci FO et al. found that lamotrigine treatment could protect the spinal cord from experimental ischemia–reperfusion injury by preventing lipid peroxidation and increased oxidative stress, as well as the consumption of antioxidant enzyme activity (Kahveci et al., 2021). It is reported that statins can reduce ischemia–reperfusion injury in many organs, including the spinal cord (Devarajan, 2006; Sironi et al., 2003). The antioxidant effect is one of the main protective mechanisms by which statins prevent neuronal death and cytotoxicity (Hwang et al., 2013). In studies related to spinal cord reperfusion injury, it has been found that statins such as atorvastatin (Kardes et al., 2017), rosuvastatin (Yavuz et al., 2013), and simvastatin (Sohn et al., 2017) can alleviate damage to spinal motor neurons caused by ischemia and reperfusion through their antioxidant effects. However, there are still significant limitations in the clinical application of the above-mentioned drugs. Firstly, the pharmacokinetic characteristics (such as half-life and tissue distribution) of dexmedetomidine and statins in the context of spinal cord injury have not been fully studied, which may affect the timing of administration and the stability of therapeutic effect. Secondly, the existence of the blood-spinal cord barrier severely limits the permeability of many drugs (such as lamotrigine) at the injury site, resulting in the actual action concentration being lower than the theoretical effective threshold. Furthermore, the dosage regimens in different studies vary greatly. For instance, there is no unified standard for the anti-inflammatory and antioxidant doses of statins, which may cause fluctuations in therapeutic efficacy or potential toxicity risks. More crucially, the existing evidence is mainly based on animal experiments, lacking large-scale clinical trials to verify the safety of these drugs in humans, their improvement effects on neurological function, and their long-term prognostic impact. In the future, spinal cord targeting needs to be enhanced by improving drug delivery systems (such as nanocarriers or targeted modifications), optimizing dosage strategies in combination with pharmacokinetic-pharmacodynamics models, and promoting multi-stage clinical trials to clarify its clinical translational value.

### 7.3 Other treatments

In addition to the above-mentioned treatments, the research also found that physical therapies such as hyperbaric oxygen and acupuncture can delay SCIRI by inhibiting oxidative damage. Studies have confirmed that hyperbaric oxygen pretreatment can induce ischemic tolerance in the brain and spinal cord (Xiong et al., 2000; Dong et al., 2002). In the rabbit SCIRI model, after pretreatment with hyperbaric oxygen, it was found that hyperbaric oxygen could activate the SOD activity in the rabbit spinal cord tissue, thereby eliminating ROS generated during ischemia–reperfusion and protecting the spinal cord from ROS-mediated injury (Nie et al., 2006). Furthermore, the research also found that acupuncture can inhibit oxidative stress to suppress the apoptosis of nerve cells, thereby delaying SCIIR (Cai and Shen, 2018). However, the clinical application of this type of physical therapy still faces many challenges. Firstly, the mechanism of action of hyperbaric oxygen and acupuncture has not been fully clarified, especially the insufficient systematic pharmacological research on their regulation of oxidative stress, making it difficult to precisely optimize the treatment plan. For example, the intervention timing, pressure parameters and treatment course standards of hyperbaric oxygen vary significantly in different studies, while the acupoint selection and stimulation intensity of acupuncture lack unified norms, which may affect the repeatability of therapeutic effects. Secondly, the targeting of physical therapy depends on local action, but the heterogeneity of the microenvironment in the spinal cord injury area may weaken its antioxidant effect. Furthermore, the existing research is mainly based on animal models and small-sample clinical trials, lacking large-scale multicenter randomized controlled trials to verify its long-term safety and the improvement effect on neurological function. In the future, it is necessary to clarify the dynamic mechanism of physical therapy through molecular imaging technology, establish individualized treatment parameters in combination with artificial intelligence algorithms, and promote evidence-based medical research to support clinical transformation.

## 8 Discussion and future outlook

The pathophysiological process after spinal cord ischemia–reperfusion injury is very complex. The imbalance of oxidants and antioxidant systems leads to an abnormal increase in ROS production. Excessive ROS can cause irreversible oxidative damage to macromolecules (such as nucleic acids, lipids and proteins), and interact with inflammatory responses, autophagy, and apoptosis. These processes can lead to complex secondary injury cascade reactions. This complexity may explain why many treatment strategies and experimental methods have not been successful in SCRI treatment. Oxidative stress plays an important role in SCIRI, and the levels of various free radicals can be used as indicators of damage. The ROS level can be regulated through the Nrf2 signaling pathway, and this regulation helps prevent or reduce spinal cord injury.

Although certain research results have been achieved on the mechanism of action and treatment strategies of oxidative stress in SCIRI at present, there are still many deficiencies. In terms of mechanism research, although it has been clearly identified that oxidative stress plays a key role in SCIRI, and certain understandings have been gained regarding the lipid peroxidation, protein oxidation, DNA damage it triggers, as well as its interaction with inflammatory responses, the potential molecular regulatory network between oxidative stress and other pathophysiological processes has not yet been fully clarified. For instance, there are relatively few studies on the interrelationships between oxidative stress and emerging research fields such as autophagy and ferroptosis in SCIRI. These complex interactions and potential molecular regulatory mechanisms still await in-depth exploration. Among the signaling pathways related to oxidative stress, although signaling pathways such as Nrf2, NF-kB and MAPK have received attention, there are still many downstream signaling molecules and the synergistic mechanisms among them that have not been fully clarified. In terms of treatment strategies, although the existing treatment methods based on oxidative stress have alleviated SCIRI to a certain extent, they still have limitations. The therapeutic effect of antioxidants is limited by factors such as the rapid generation rate of free radicals, drug stability and low bioavailability. It is difficult to completely eliminate excessive ROS in the body, and the optimal dosage, time window and combined use regimens of different antioxidants still need to be further optimized. Drugs that regulate oxidative stress signaling pathways face safety and side effect issues in clinical applications, and their long-term efficacy and potential risks also need to be verified by more large-scale clinical trials. The mechanisms of action of hyperbaric oxygen therapy and acupuncture therapy have not been fully clarified yet. There is a lack of unified treatment standards and norms, and the individual differences in treatment effects are relatively large.

In the future, in-depth research on the molecular mechanism of oxidative stress in SCIRI remains key. On the one hand, research on the interaction between oxidative stress and other pathophysiological processes should be strengthened. For instance, the relationship between oxidative stress and autophagy, ferroptosis, neuroplasticity, etc. should be deeply explored to reveal its potential molecular regulatory network and provide a theoretical basis for finding new therapeutic targets. Using multi-omics techniques, such as transcriptomics, proteomics, and metabolomics, etc., the changes of genes, proteins and metabolites during the SCIRI process were comprehensively analyzed to screen out the key molecules and signaling pathways closely related to oxidative stress. On the other hand, in terms of treatment strategies, efforts should be made to develop more effective treatment methods. Develop new types of antioxidants to enhance their stability, bioavailability and antioxidant activity, explore the application of emerging technologies such as nanotechnology and gene therapy in antioxidant treatment, and achieve precise delivery and targeted therapy of antioxidants. For example, through gene editing technology, the expression of antioxidant enzymes in spinal cord tissue is upregulated to enhance the body’s own antioxidant capacity. Using nanocarriers to precisely deliver antioxidants or drugs that regulate oxidative stress signaling pathways to damaged spinal cord tissue, improving therapeutic efficacy and reducing side effects. Further optimizing existing treatment methods and developing personalized treatment plans are also crucial. Based on the individual differences of patients, such as age, underlying diseases, and the degree of injury, optimize the regimens of antioxidant therapy, hyperbaric oxygen therapy, and acupuncture treatment to enhance the pertinence and effectiveness of the treatment. Strengthen research on combination therapy strategies, explore the synergistic mechanisms between different treatment methods, such as the combined application of antioxidants and stem cell therapy, neurotrophic factor therapy, etc., to provide more effective options for the clinical treatment of SCIRI. Strengthen the transformation between basic research and clinical practice, apply laboratory research results to clinical practice as soon as possible, carry out more large-scale and multi-center clinical trials, verify the safety and effectiveness of treatment methods, and bring better therapeutic effects and quality of life improvements to patients with SCIRI.

## Glossary

SCIRI: Spinal cord ischemia/reperfusion injury
ROS: Reactive oxygen
BSCB: Blood-spinal cord barrier
O_2_^-^: Superoxide anion
H_2_O_2_: Hydrogen peroxide
OH^−^: hydroxyl radical
I/R: Ischemia reperfusion
ETC: Electron transport chain
SOD: superoxide dismutase
XDH: xanthine dehydrogenase
XO: Xanthine oxidase
NADPH: Phosphamide adenine dinucleotide
PRDX2: Peroxidase 2
mPTP: Endometrial permeability transition pore
ΔΨm: membrane potential
Cyt C: Cytochrome C
GSDMD: gasdermin-D
Apaf-1: Apoptotic protease activator-1
mtROS: Mitochondrial reactive oxygen species
HIF-1α: Hypoxia-inducible factor-1α
GSH: Glutathione
GPX4: Glutathione peroxidase 4
Nrf-2: Nuclear factor erythrocyte 2-related factor 2
ARE: Antioxidant response element
Keap1: Kelch-like epichlorohydrin-associated protein-1
HO-1: Heme oxygenase-1
NQO1: Quinone oxidoreductase-1
GPX: glutathion peroxidase
NF-κB: Nuclear factor-κB
MDA: Malonaldehyde
RIPC: Remote ischemic preconditioning
MAPK: mitogen activated protein kinase
MMPs: Matrix metalloproteinase
ERK: Extracellular signal-regulated kinase
JNK: c-Jun NH 2-terminal kinase
Sal: salidroside
BA: Biochanin
AST: astaxanthin
GRb1: Ginsenoside Rb1
MAP3K: APK kinase kinases
