# Correction: The role of oxidative stress in spinal cord ischemia reperfusion injury: mechanisms and therapeutic implications

**DOI:** 10.3389/fncel.2025.1742278

**Published:** 2025-11-20

**Authors:** Yu Xing, Yuan-zhang Xiao, Min Zhao, Jiang-jun Zhou, Kai Zhao, Chun-lin Xiao

**Affiliations:** 1 Department of Orthopaedics, First Affiliated Hospital of Gannan Medical University, Ganzhou, Jiangxi, China; 2 Gannan Medical University, 1 Harmony Avenue, Rongjiang New District, Ganzhou, Jiangxi Province, China; 3 Department of Spine Surgery, Yingtan People's Hospital, Yingtan, Jiangxi, China; 4 Hospital 908, Joint Logistics Support Force, Nanchang, Jiangxi, China

**Keywords:** spinal cord ischemia/reperfusion injury (SCIRI), oxidative stress, inflammation, cell death, mechanism

In the published article, there was a mistake in the designation of co-first authors. The author list was erroneously given as “Yu Xing^1, 2^, Yuan-zhang Xiao^1, 2†^, Min Zhao^3^, Jiang-jun Zhou^4^, Kai Zhao^1, 2*†^ and Chun-lin Xiao^1,2^^*^”, where the equal contribution symbol “†” was incorrectly placed next to Kai Zhao's name, rather than Yu Xing's name. The corrected author list appears below:

Yu Xing^1, 2†^, Yuan-zhang Xiao^1, 2†^, Min Zhao^3^, Jiang-jun Zhou^4^, Kai Zhao^1,2^^*^ and Chun-lin Xiao^1,2*^.

The original version of this article has been updated.

